# GPER mediates differential effects of estrogen on colon cancer cell proliferation and migration under normoxic and hypoxic conditions

**DOI:** 10.18632/oncotarget.20653

**Published:** 2017-09-06

**Authors:** Viviana Bustos, Áine M. Nolan, Anke Nijhuis, Harry Harvey, Alexandra Parker, Richard Poulsom, Jean McBryan, Warren Thomas, Andrew Silver, Brian J. Harvey

**Affiliations:** ^1^ Department of Molecular Medicine, Education and Research Centre, Beaumont Hospital, Royal College of Surgeons in Ireland, Dublin, Ireland; ^2^ Centre for Digestive Diseases, National Centre for Bowel Research and Surgical Innovation, Blizard Institute, Barts and The London School of Medicine and Dentistry, London, UK

**Keywords:** estrogen, GPER, VEGF, hypoxia, colorectal cancer

## Abstract

The estrogen receptor ERβ is the predominant ER subtype expressed in normal well-differentiated colonic epithelium. However, ERβ expression is lost under the hypoxic microenvironment as colorectal cancer (CRC) malignancy progresses. This raises questions about the role of signalling through other estrogen receptors such as ERα or G-protein coupled estrogen receptor (GPER, GPR30) by the estrogen 17β-estradiol (E2) under hypoxic conditions after ERβ is lost in CRC progression. We tested the hypothesis that E2 or hypoxia can act via GPER to contribute to the altered phenotype of CRC cells.

GPER expression was found to be up-regulated by hypoxia and E2 in a panel of CRC cell lines. The E2-modulated gene, Ataxia telangiectasia mutated (*ATM*), was repressed in hypoxia via GPER signalling. E2 treatment enhanced hypoxia-induced expression of HIF1-α and VEGFA, but repressed HIF1-α and VEGFA expression under normoxic conditions. The expression and repression of VEGFA by E2 were mediated by a GPER-dependent mechanism. E2 treatment potentiated hypoxia-induced CRC cell migration and proliferation, whereas in normoxia, cell migration and proliferation were suppressed by E2 treatment. The effects of E2 on these cellular responses in normoxia and hypoxia were mediated by GPER. In a cohort of 566 CRC patient tumor samples, *GPER* expression significantly associated with poor survival in CRC Stages 3-4 females but not in the stage-matched male population.

Our findings support a potentially pro-tumorigenic role for E2 in ERβ-negative CRC under hypoxic conditions transduced via GPER and suggest a novel route of therapeutic intervention through GPER antagonism.

## INTRODUCTION

A higher incidence of colorectal cancer (CRC) is found in males compared to females [1], and young women (18-44 years) with CRC have a better survival outcome compared to men of the same age [2] or to older women (over 50 years) [3]. These data suggest a protective role for the steroid hormone estrogen in CRC development. However, this premise remains controversial as recent epidemiological studies indicated that hormone replacement therapy (HRT) in post-menopausal women did not confer a protective effect [4, 5] in contrast to previous findings [6, 7]. The role of estrogen and estrogen receptors in the onset and progression of CRC is not well understood. Estrogen may have a protective effect conferred through full-length ERβ (ERβ1) expression [8]. ERβ1 is the predominant ER in the differentiated colonic epithelium and sustained ERβ1 expression in CRC correlates with better prognosis and patient survival. As the malignancy progresses ERβ1 is lost correlating with worse prognosis [8, 9]. The relative expression levels of ER isoforms may complicate the interpretation of the Women's Health Initiative findings on protective/exacerbating effects of estrogen in CRC [5, 7].

ERβ1 over-expression in CRC cells can also result in decreased cell migration [10], but whether estrogen can promote tumor progression following the loss of ERβ is unknown. E2-mediated promotion of tumor progression after ERβ loss may be through other estrogen-ligand receptors such as ERα or G protein-coupled estrogen receptor (GPER, GPR30) activation, as has been reported in breast cancer [11].

The influence of the CRC tumor microenvironment on the tumorigenic role of estrogen is unknown. Hypoxic (≤3% oxygen) and anoxic (≤0.3% oxygen) cancer cells display increased proliferation, increased angiogenesis and metastatic drive, and therapy resistance [12], due in part to the induction and activation of the hypoxia-inducible transcription factor HIF1-α [12]. In CRC, over-expression of HIF1-α and the HIF target vascular endothelial growth factor (VEGF), are independently associated with poor CRC patient survival [13, 14]. Interestingly, *GPER* is a direct HIF1-α target and is up-regulated following HIF stabilization in breast cancer cells [16].

Tumor cells show altered expression of key DNA damage repair genes such as ataxia telangiectasia mutated (*ATM*) [15], which drives the proliferation of genetically unstable tumor cells [16]. Loss of *ATM* expression is associated with poor survival in CRC [17]. In cervical cancer cells loss of ATM correlates with *HIF* expression [18]. An increase in phosphorylated ATM levels in hypoxic HCT116 colon cancer cells was described [19], but the modulation of *ATM* expression by low oxygen tension and the sensitivity of expression to E2, in CRC was not investigated. Hypoxia and estrogen are functionally equivalent in breast cancer cells [20] and E2 induces an increase in both HIF1A and VEGF gene expression [21, 22]. In contrast, VEGF is repressed by ERβ1 over-expression in HT-29 colon cancer cells [23]. The induction and/or repression of ERα [24, 25], ERβ [24] and *GPER* [26] have also been reported in breast cancer cells. However, the functional consequences of E2 action within the hypoxic CRC cell micro-environment have not been investigated.

Here, we present novel insights into the protective or exacerbating effects of E2 on CRC tumor biology modulated by oxygen tension associated with the tumor microenvironment.

## RESULTS

### HT-29 CRC cells are oxygen-sensitive

**Figure 1:**
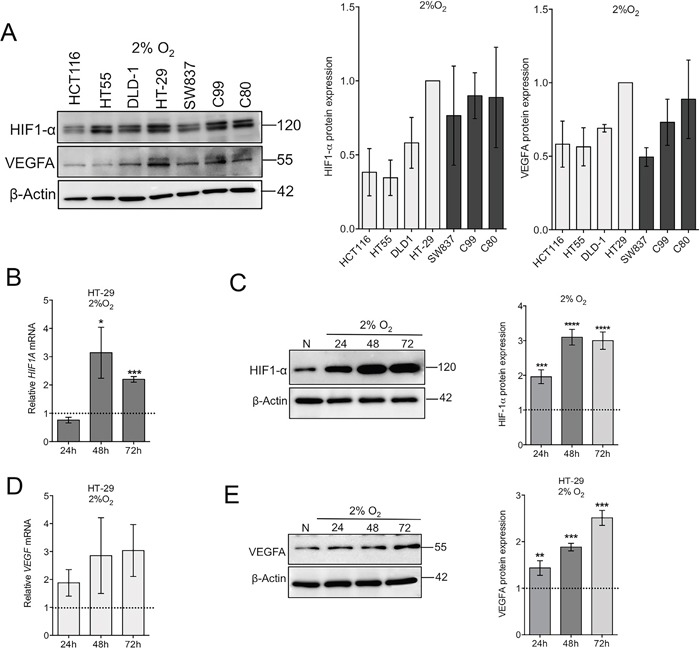
Hypoxic sensitivity of colorectal cancer cell lines **(A)** Western blot and densitometry analysis of HIF1-α and VEGFA protein expression in a panel of colon (HCT116, HT55, DLD-1 and HT-29) and rectal (SW837, C99 and C80) cancer cell lines cultured in 2% oxygen for 24h. n=4, error bars represent SEM. **(B)** Time course of *HIF1A* mRNA expression in HT-29 colon cancer cells in response to culturing in 2% oxygen for 24, 48 and 72h. The dotted line represents basal expression under normoxic conditions. Mean ± SEM, n=3-4. **(C)** Western blot and densitometry analysis of HIF1-α protein expression in response to hypoxic culture. Mean ± SEM, n=4. **(D)** Time course of *VEGF* mRNA expression in HT-29 cells under hypoxic conditions. The dotted line represents basal expression under normoxic conditions. Mean ± SEM, n=3-4. **(E)** Western blot and densitometry analysis of VEGFA protein expression in response to hypoxic culture. Mean ± SEM, n=4. mRNA levels were normalized to *PPIB* endogenous control. β-actin was used as a loading control for protein. ^*^P<0.05, ^**^P<0.01, ^***^P<0.001, ^****^P<0.0001. *P*-values relative to normoxic controls.

**Figure 2:**
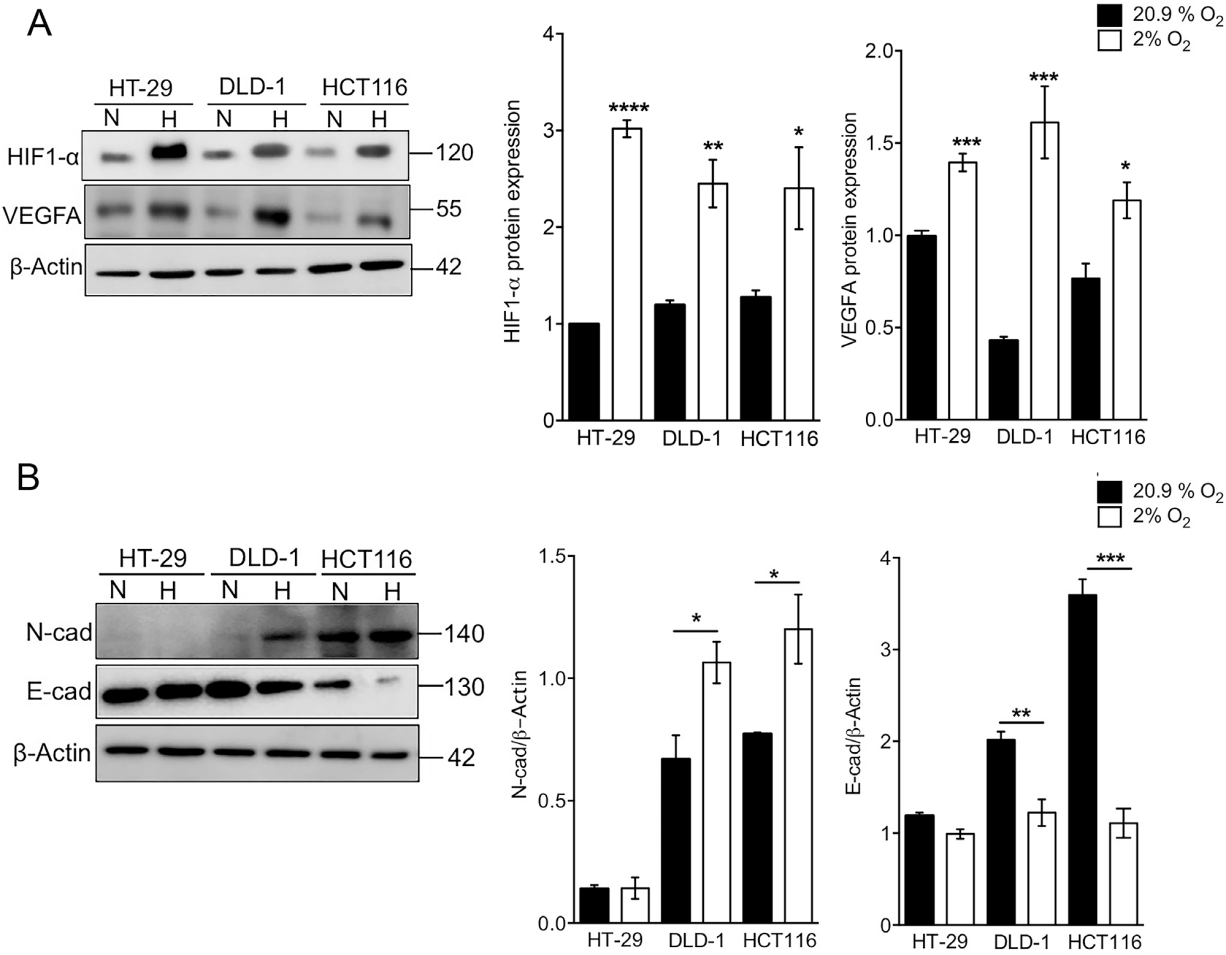
Hypoxic effects on differentiation status of colon cancer cells **(A)** Western blot and densitometry analysis showing increased HIF1-α and VEGFA protein following 24h culture in hypoxia (H) compared to normoxia (N) in a panel of colon cancer cell lines (HT-29, DLD-1 and HCT116). β-actin was used as a loading control. Expression was normalised to the protein expression under normoxic conditions in HT-29 cell line. Mean ± SEM, n=4-5 ^**^P<0.001, ^***^P<0.0001, ^****^P<0.00001. **(B)** Western blot and densitometry analysis of E-cadherin and N-cadherin protein expression. Hypoxia promotes N-cadherin and further suppresses E-cadherin expression in cells exhibiting a moderately and less differentiated epithelial phenotype (DLD-1 and HCT116 respectively). HT-29 cells display an epithelial phenotype in both normoxia and hypoxia. Mean ± SEM, n=4. ^*^P<0.01, ^**^P<0.001, ^***^P<0.0001.

We investigated whether CRC cell lines exhibit a typical HIF-1α expression in response to low oxygen tension, including induction of HIF-1α-responsive genes such as *VEGF* [27]. In a panel of six CRC cell lines (colon cancer: HT-29, DLD-1, HT55, and HCT116; rectal cancer: C80 and C99), HIF-1α and VEGF protein levels were detected in all cells cultured under hypoxic conditions (2% oxygen) for 24h (Figure 1A), however the biggest response was observed in HT-29 cells and was used as the reference model in subsequent experiments. In the HT-29 colon cancer cells, hypoxia induced a 3-fold increase in HIF-1α protein and mRNA expression with protein levels increasing after only 24 hours hypoxia (Figure 1B and 1C). VEGFA expression also increased at both the mRNA and protein levels, further confirming the hypoxic response of HT-29 cells to low oxygen tension (Figure 1D and 1E). HT-29 cells are recognised as a well-differentiated colon cancer cell line [28]. By comparison, HCT116 cells are poorly differentiated and DLD-1 colon cells have an intermediate phenotype [29] but their phenotypic responses to hypoxia are unknown. All three cell lines exhibited a hypoxic response to 24h culture in 2% oxygen as evidenced by increases in HIF-1α and VEGFA protein expression (Figure 2A). HT-29 cells express high levels of E-cadherin and low levels of N-cadherin and these differentiation characteristics were not affected by hypoxia (Figure 2B). In contrast, DLD-1 and HCT116 cells underwent de-differentiation in response to hypoxia, with increased N-cadherin and decreased E-cadherin expression detectable following culture under hypoxic conditions (Figure 2B).

### Estrogen induces GPER expression in normoxia and hypoxia

**Figure 3:**
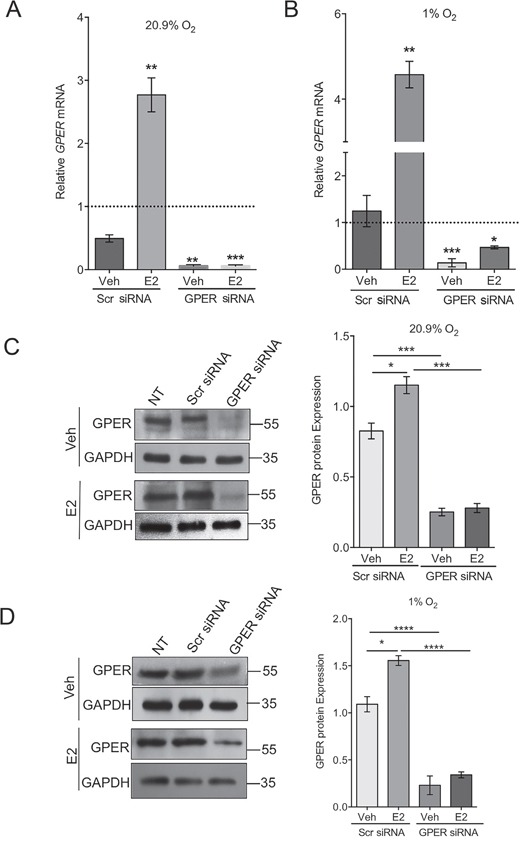
Estrogen increases GPER expression in normoxia and hypoxia **(A, B)** Real-time PCR analysis of *GPER* mRNA expression normalised to 18S rRNA in transfected HT-29 cells treated with ethanol (Veh) or 10nM Estradiol (E2) for 24h. The experiment was conducted under normoxic (A) and hypoxic (B) conditions. Cells were transfected with either scrambled control (Scr) or GPER-targeting siRNA. The dotted line represents basal expression in non-transfected, untreated cells. (Mean ± SEM, n=3). P-values are relative to the Veh Scr siRNA control. ^*^P<0.05, ^**^P<0.01, ^***^P<0.001. **(C, D)** Western blot and densitometry analysis of GPER protein expression, normalised to GAPDH, in transfected HT-29 cells treated with ethanol (Veh) or 10nM Estradiol for 24h. The experiment was conducted under normoxic (C) and hypoxic (D) conditions. Mean ± SEM, n=4. ^*^P<0.01, ^***^P<0.001, ^****^P<0.0001.

We were unable to detect ERα and ERβ mRNA expression in HT-29 cells (data not shown). Under normoxic conditions, HT-29 cells showed the highest basal expression of GPER under hypoxic conditions (Supplementary Figure 1B) and are thus the most relevant and suitable model to investigate the effects of GPER knock-down on function. The predicted molecular weight for GPER is 42kDa, however, this refers to the purified recombinant GPER and not the endogenous protein expressed in cells which can undergo extensive glycosylation. Higher molecular weight sizes of GPER between 50 and 60 kDa have been reported due to glycosylation and interaction with other proteins. We detected GPER as a strong band at 55kDa and have provided a control to show that this band is GPER by establishing knockdown cells using siRNA targeting GPER in which this band at 55kDa is lost (Figure 3 and Supplementary Figure 3).

Both the mRNA and protein levels of GPER were increased in response to 24h estrogen treatment (Figure 3A and 3C). Under hypoxic conditions, GPER expression was increased in HT-29 cells and was notably higher than GPER expression in other CRC cell lines (Supplementary Figure 1). Estrogen treatment under hypoxic conditions produced a similar response as was observed under normoxia with increases in both mRNA and protein expression of GPER (Figure 3). A siRNA targeting GPER was used successfully to decrease the mRNA and protein expression of GPER (Figure 3). In the presence of this siRNA, estrogen failed to increase GPER protein expression significantly under normoxic or hypoxic conditions (Figure 3A-3D).

### Estrogen suppresses ATM expression via GPER in normoxia and hypoxia

**Figure 4:**
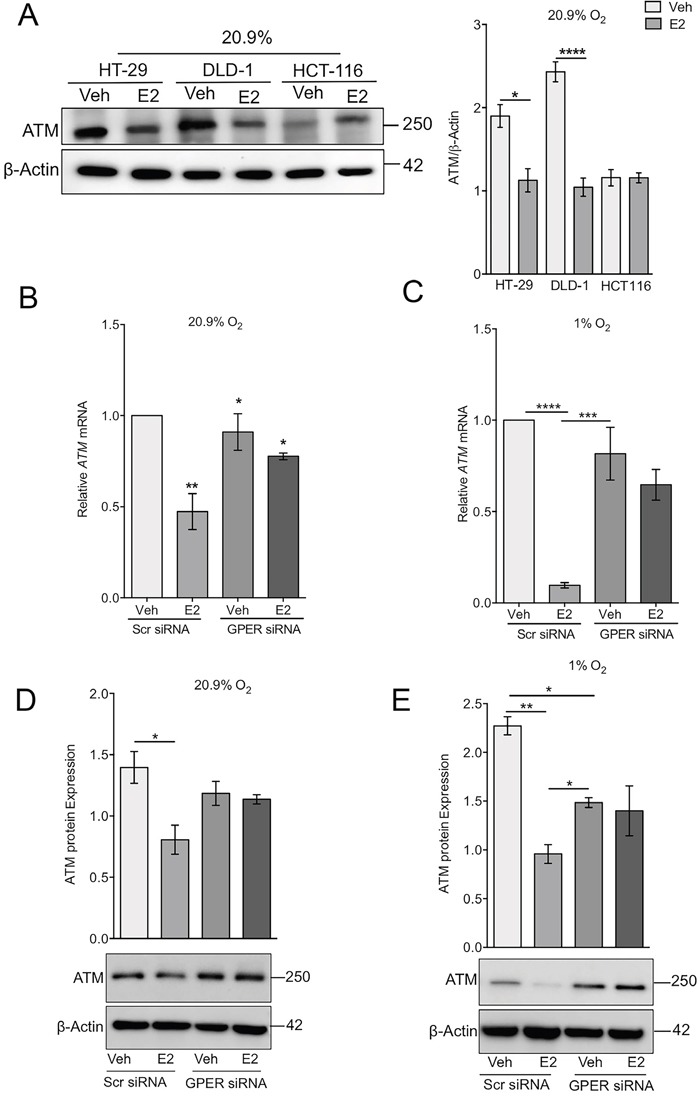
GPER-dependent regulation of ATM expression by estrogen **(A)** Western blot and densitometry analysis of ATM protein expression in a panel of cell lines (HT-29, DLD-1 and HCT116) treated with ethanol control (Veh) or 10nM estradiol (E2) under normoxic conditions. β-actin was used as a loading control, mean ± SEM, n=4, ^*^P<0.01, ^****^P<0.0001. **(B, C)** Real-time PCR analysis of *ATM* mRNA normalised to 18S rRNA in HT-29 cells transfected with scrambled siRNA (Scr) or GPER Silencer selected RNA (GPER siRNA). Cells were treated with ethanol (Veh) or 10nM estradiol (E2) for 24h. Cells were cultured in either normoxic conditions (B) or with 2% oxygen (C). Mean ± SEM, n=4, P-values are relative to normoxic vehicle treated conditions, ^*^P<0.05, ^**^P<0.01;^***^P<0.001 ^****^P<0.0001. **(D, E)** Western blot and densitometry of ATM protein expression from the same experiments conducted in (B) and (C). Mean ± SEM, n=4, ^*^P<0.05, ^**^P<0.01.

The DNA repair gene ATM has been associated with a protective effect in CRC and loss of ATM has been correlated with poor outcome in CRC patients [21]. To investigate the impact of estrogen on ATM expression, HT-29, DLD-1 and HCT116 cells were treated with estrogen for 24h under normoxic conditions. Estrogen repressed ATM protein expression in both HT-29 and DLD-1 cells significantly, but not in the less differentiated HCT116 cells (Figure 4A). Moreover, the repression of ATM caused by estrogen in HT-29 cells was confirmed to be via GPER as siRNA targeting GPER silenced the estrogenic effects on ATM expression at both the mRNA and protein levels (Figure 4B and 4D). Hypoxia also induced repression of ATM in HT-29 and DLD-1 cells (Supplementary Figure 2A). Under hypoxic conditions, estrogen further suppressed ATM expression and this was confirmed to involve a GPER-dependent mechanism since the ATM response to estrogen was lost in GPER-silenced cells (Figure 4C and 4E). The GPER agonist G1 also produced a decrease in ATM mRNA expression under normoxic conditions (Supplementary Figure 2B).

### Estrogen, acting via GPER, differentially regulates VEGFA and HIF-1α in normoxia and hypoxia

**Figure 5:**
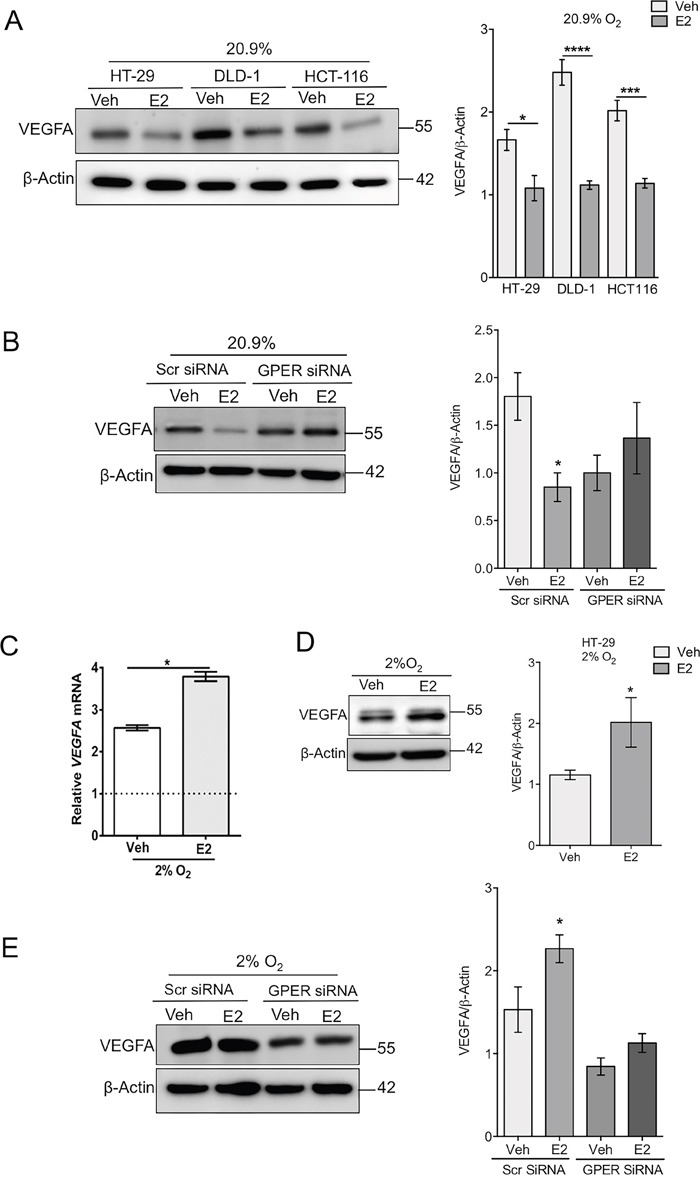
GPER- and oxygen-dependent regulation of VEGF expression by estrogen **(A)** Western blot and densitometry analysis of VEGFA protein expression in a panel of cell lines (HT-29, DLD-1 and HCT116) treated with ethanol control (Veh) or 10nM estradiol (E2) under normoxic conditions. β-actin was used as a loading control. Mean ± SEM, n=4, ^*^P<0.01, ^***^P<0.001, ^****^P<0.0001, p-values relative to vehicle treated controls. **(B)** Western blot and densitometry of VEGFA protein expression in normoxic transfected HT-29 cells treated with vehicle or estradiol. Scrambled control (Scr) or GPER-targeting siRNA were used. Mean ± SEM, n=4, ^*^P<0.05, p-value relative to vehicle treated control. **(C)** *VEGF* mRNA expression normalised to *PPIB* endogenous control in HT-29 cells treated with ethanol (Vehicle) or 10nM estradiol (E2) under hypoxic conditions (2% O_2_). Dotted line represents vehicle-treated normoxic HT-29 cells. Mean ± SEM, n=3, ^*^P<0.01, p-value relative to vehicle treated control. **(D)** Western blot and densitometry analysis of VEGF protein expression in hypoxic HT-29 cells treated with ethanol (Veh) or 10nM estradiol (E2). β-actin was used as a loading control, mean ± SEM, n=5 ^*^P<0.05. p-value relative to vehicle treated control. **(E)** Western blot and densitometry of VEGFA protein expression in hypoxic transfected HT-29 cells treated with vehicle or estradiol. Scrambled control (Scr) or GPER-targeting siRNA were used. Mean ± SEM, n=5 ^*^P<0.05. P-value relative to vehicle Scr treated control.

**Figure 6:**
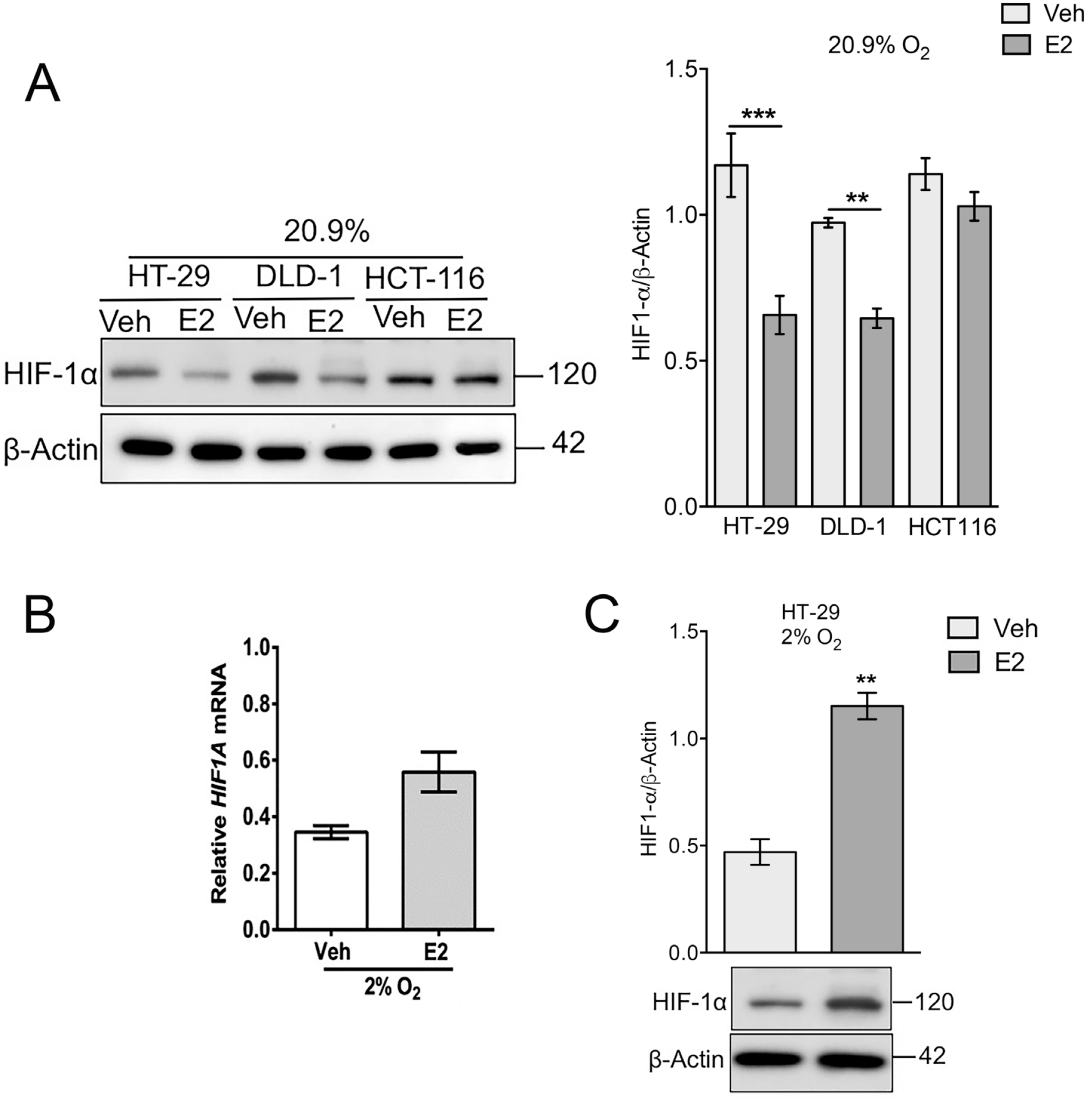
Oxygen tension and estradiol modulate HIF1-α expression in colon cancer cells **(A)** Western blot and densitometry analysis of HIF1-α protein expression in a panel of cell lines (HT-29, DLD-1 and HCT116) treated with ethanol control (Veh) or 10nM estradiol (E2) under normoxic (20.9% O_2_) conditions. β-actin was used as a loading control. Mean ± SEM, n=4, ^*^P<0.01, ^***^P<0.001, p-values relative to vehicle treated controls. **(B)** *HIF1A* mRNA expression normalised to *PPIB* endogenous control in HT-29 cells treated with ethanol (Veh) or 10nM estradiol (E2) under hypoxic conditions (2% O_2_). Dotted line represents vehicle-treated normoxic HT-29 cells. Mean ± SEM, n=3. **(C)** Western blot and densitometry analysis of HIF1-α protein expression in hypoxic HT-29 cells treated with ethanol (Veh) or 10nM estradiol (E2). β-actin was used as a loading control, mean ± SEM, n=4 ^*^P<0.05. P-value relative to vehicle treated control.

Over-expression of VEGFA and HIF-1α are independently associated with poor outcome in CRC [13, 14]. In normoxic HT-29 cells, estrogen treatment suppressed VEGFA and HIF-1α expression (Figure 5A and Figure 6). The regulation of VEGFA was confirmed to be dependent on GPER since the suppression of VEGFA by estrogen was diminished in GPER-silenced cells (Figure 5B). However, in stark contrast, under hypoxic conditions, estrogen increased VEGFA expression at both the mRNA and protein levels (Figure 5C and 5D). Moreover, a GPER-dependence of the stimulatory effects of estrogen on VEGFA was observed under hypoxic conditions (Figure 5E). Estrogen also significantly increased HIF-1α protein levels under hypoxic conditions at the 24h time point examined (Figure 6). This is a novel finding whereby estrogen, acting through GPER, can produce potentially tumor-suppressing effects in normoxia but opposing, tumor-promoting effects under hypoxic conditions and may offer a molecular mechanism to explain the apparent contradictory observations in patient studies on sexual dimorphism and estrogen protection/exacerbation in CRC. If the oxygen tension in the tumor microenvironment can modulate the estrogen effects on GPER and VEGF this could provide a mechanism for opposing effects of estrogen depending on the CRC stage and the site of sampling from tumor biopsies.

### Estrogen, acting via GPER, suppresses migration in normoxia but enhances it in hypoxia

**Figure 7:**
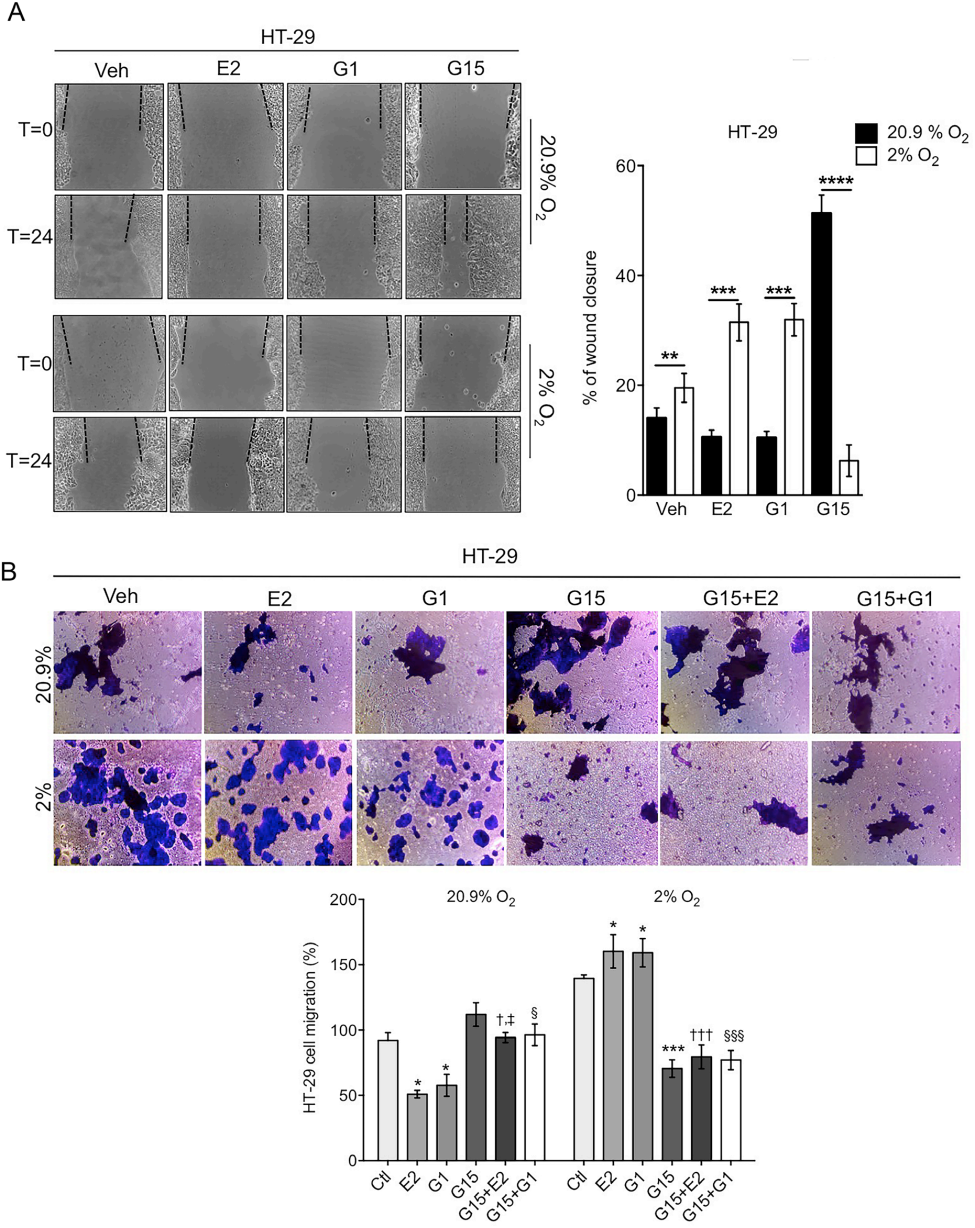
Oxygen-dependent regulation of migration by estrogen in HT-29 cells **(A)** Wound healing assay in HT-29 cells under normoxic (20.9% O_2_) and hypoxic (2% O_2_) conditions. Representative images are shown of the scratches at time 0 and following 24h. Dashed lines indicate wound margins. Graph shows the % wound closure under each condition. Cells were treated with 10nM estradiol (E2), 1μM G1 (a GPER-selective agonist) or 5μM G15 (a GPER-selective antagonist). Mean ± SEM, n=6, ^**^P<0.01, ^***^P<0.001, ^****^P<0.0001. **(B)** Boyden chamber migration assay in HT-29 cells under normoxic and hypoxic conditions. Representative images are shown. Cells were treated with E2, G1, G15, G15+E2 or G15+G1. Mean ± SEM, n=6, ^*^P<0.01 E2 and G1 treatment compared to Ctl (Veh only), †*P*<0.01 G15+E2 compared to E2 treatment, ‡*P*<0.01 G15+E2 compared to G15 treatment, §*P*<0.01 G15+G1 compared to G1 treatment, ^***^P<0.0001 G15 compared to E2 and G1, †††*P*<0.001 G15+E2 treatment compared to E2 treatment, §§§*P*<0.0001 G15+G1 compared to G1 treatment.

**Figure 8:**
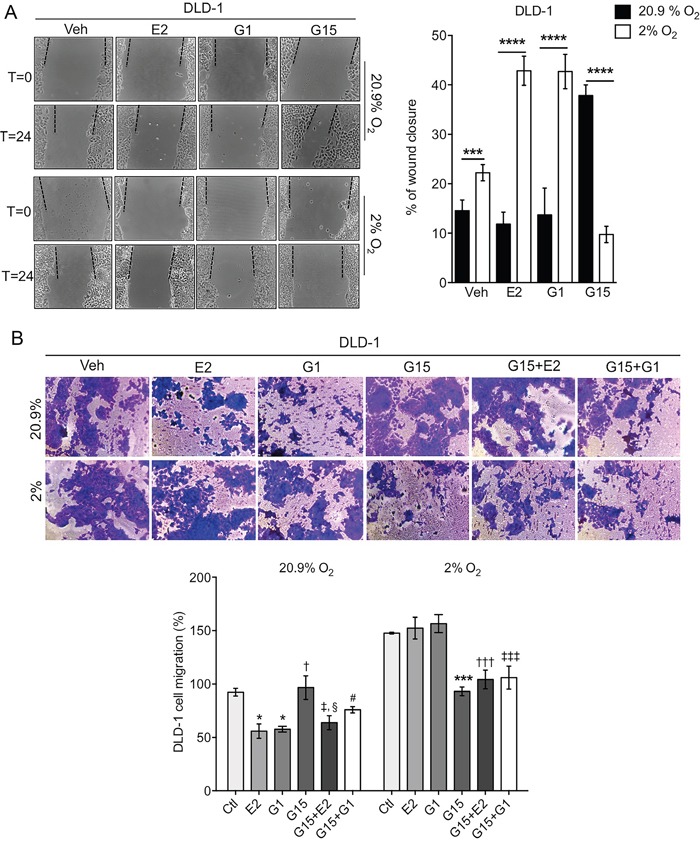
Oxygen-dependent regulation of wound healing and migration by estradiol in DLD-1 cells **(A)** Wound healing assay in DLD-1 cells under normoxic (20.9% O_2_) and hypoxic (2% O_2_) conditions. Representative images are shown of the scratches at time 0 and following 24h. Dashed lines indicate wound margins. Graph shows the % wound closure under each condition. Cells were treated with 10nM estradiol (E2), 1μM G1 (a GPER-selective agonist) or 5μM G15 (a GPER-selective antagonist). Mean ± SEM, n=6, ^***^P<0.001, ^****^P<0.0001. **(B)** Boyden chamber migration assay in DLD-1 cells under normoxic and hypoxic conditions. Representative images are shown. Cells were treated with E2, G1, G15, G15+E2 or G15+G1. Mean ± SEM, n=6, ^*^P<0.01 E2 and G1 treatment compared to Ctl (Veh only), †*P*<0.01 G15 compared to G1 and E2 treatment, ‡*P*<0.01 G15+E2 compared to G15 treatment, §*P*<0.01 G15+E2 compared to E2 and G1 treatment, #*P*<0.01 G15+G1 compared to G15, ^***^P<0.0001 G15 compared to E2 and G1, †††*P*<0.001 G15+E2 treatment compared to E2 or G1 treatments, ‡‡‡*P*<0.0001 G15+G1 compared to E2 or G1 treatments.

To investigate if estrogen and GPER have functional roles to play in CRC cells, two types of migration assay were used. Both the scratch wound and Boyden chamber assays produced the same pattern of estrogen modulation of CRC cell migration – stimulation under hypoxic conditions and inhibition of migration under normoxic conditions in HT-29 (Figure 7) and DLD-1 (Figure 8) CRC cells. Under normoxic conditions, estrogen, or the GPER-selective agonist G1, reduced cell migration. Consistent with this, G15, an antagonist of GPER, enhanced migration of both HT-29 and DLD-1 CRC cells and limited the estrogenic suppression of migration (Figure 7 and Figure 8).

Basal levels of migration were significantly enhanced in both HT-29 and DLD-1 cells under hypoxic compared to normoxic conditions (Figure 7 and Figure 8). In contrast to the normoxic response, both estrogen and G1 enhanced cell migration in hypoxic conditions. This response was inhibited by the GPER antagonist G15 (Figure 7 and Figure 8). Thus, the protective effect of reduced migration with estrogen in normoxia was lost under reduced oxygen conditions.

**Figure 9:**
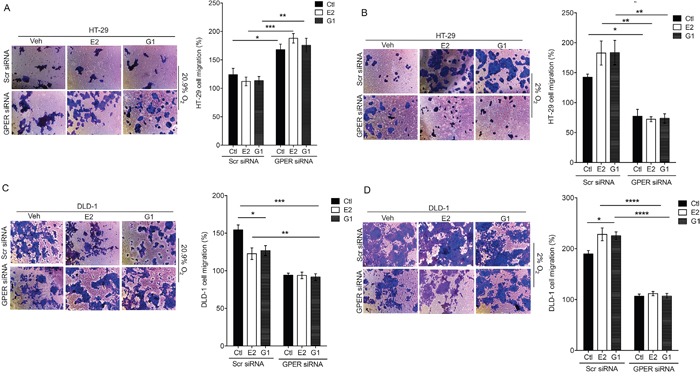
GPER-dependent regulation of migration by estrogen in HT-29 cells Boyden chamber migration assay in transfected HT-29 and DLD-1 cells under normoxic **(A, C)** and hypoxic **(B, D)** conditions. Cells were transfected with control scrambled siRNA (Scr) or GPER-targeting siRNA (GPER siRNA). Cells were treated with ethanol (Veh), 10nM estradiol (E2) or 1μM G1 for 24h. Graph shows % cell migration. Mean ± SEM, n=6, ^*^P<0.05, ^**^P<0.01, ^***^P<0.001, ^****^P<0.0001.

To further confirm the involvement of GPER in the cell migration responses to estrogen, the Boyden chamber assay was repeated using siRNA knock-down of GPER (Figure 9 and Supplementary Figure 3). The silencing of GPER expression led to significantly enhanced basal migration under normoxic conditions and reduced migration under hypoxic conditions in HT-29 cells (Figure 9A and 9B) and in DLD-1 cells (Figure 9C and 9D). Under normoxic conditions the ability of estrogen and G1 to retard cell migration was lost in GPER-silenced cells (Figure 9A and 9C). Conversely, the effects of estrogen and G1 to increase cell migration in hypoxia were lost in GPER-silenced cells (Figure 9B and 9D).

### Estrogen, acting via GPER, suppresses proliferation in normoxia but enhances it in hypoxia

**Figure 10:**
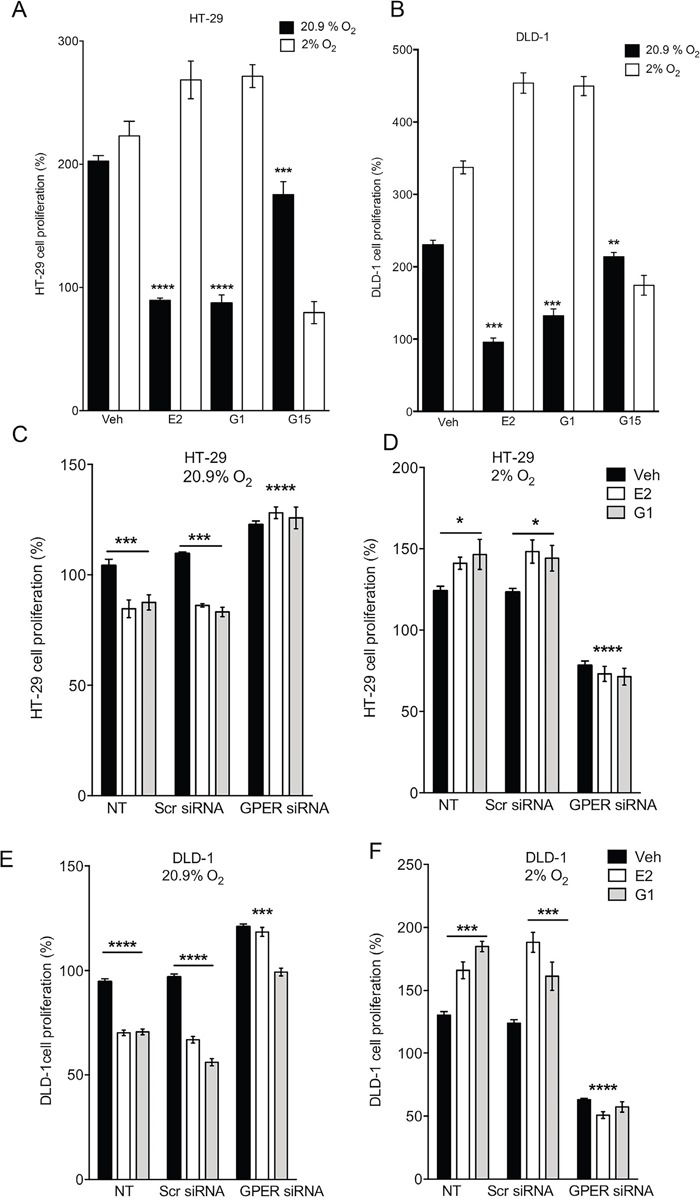
Oxygen- and GPER- dependent regulation of proliferation by estrogen in HT-29 cells **(A, B)** Proliferation assay in normoxic and hypoxic HT-29 (A) and DLD-1 (B) cells treated with E2 (10nM), G1 (1μM) and/or G15 (5 μM) for 48h. Mean ± SEM, n=15. ^***^P<0.001, ^****^P<0.0001. **(C, D)** Proliferation assay in normoxic HT-29 (C) and DLD-1 (D) transfected cells treated with Veh, E2 or G1. Cells were transfected with control scrambled or GPER-targeting siRNA. **(E, F)** Proliferation assay in hypoxic HT-29 (E) and DLD-1 (F) transfected cells treated with Veh, E2 or G1. Cells were transfected with control scrambled (Scr) or GPER-targeting siRNA. Mean ± SEM, n=12, ^*^P<0.01, ^***^P<0.001, ^****^P<0.0001.

To further explore the pro- and anti-tumorigenic potential of estrogen, proliferation assays were conducted with HT-29 (slow proliferative phenotype) and DLD-1 cells (fast proliferative phenotype) (Figure 10). Under normoxic conditions, estrogen and G1 both suppressed proliferation (Figure 10A and 10B). Under hypoxic conditions, the estrogenic signal produced the opposite functional effect with both estrogen and G1 enhancing proliferation and G15 inhibiting proliferation (Figure 10A and 10B). Under both normoxic and hypoxic conditions, the knock-down of GPER expression using siRNA prevented estrogen and G1 from altering cell proliferation rates (Figure 10C-10F). Thus, GPER is essential in transducing the normoxic anti-proliferative effects of estrogen as well as the hypoxic proliferative effects of estrogen.

### GPER expression is associated with poor CRC patient outcome

**Figure 11:**
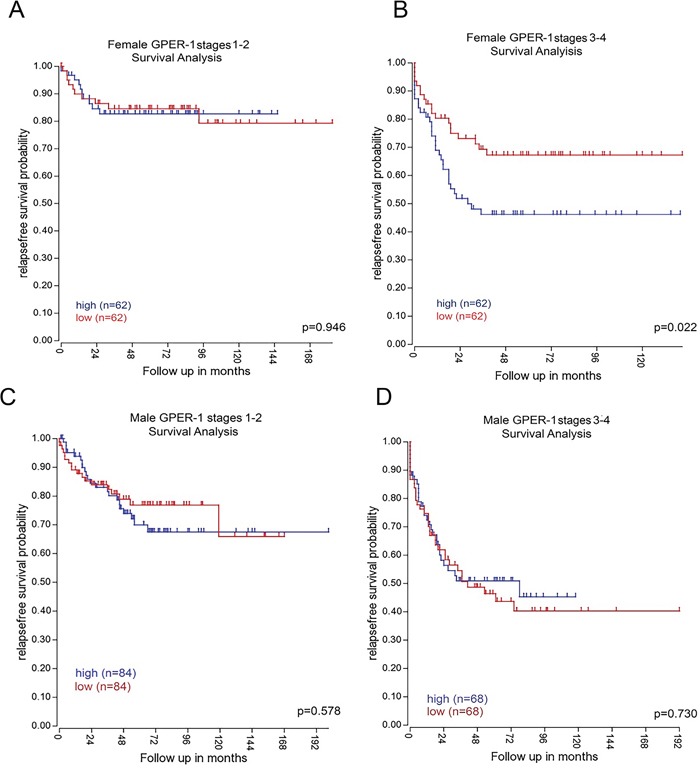
Association of GPER with survival in CRC patients Kaplan-Meier analysis of relapse-free survival from 566 colon cancer patients. Expression of GPER mRNA using a median cutoff in female patients at stage 1-2 **(A)** and at stage 3-4 **(B)** and in male patients stages 1-2 **(C)** and stages 3-4 **(D)**. Female patients with high GPER had significantly worse survival rates than those with low GPER in the late stages 3-4 (*P*=0.022).

The potential clinical relevance of GPER was analysed by Kaplan Meier analysis of a published microarray data set of 566 patients. Substratification of tumors based on patient gender and tumor stage at diagnosis was also carried out. Using a median cut-off, high expression of GPER significantly associated with poor relapse free survival in women with stages 3 and 4 CRC (P=0.022, Figure 11B). Of note, there was no significant difference in survival for male or female patients with stage 1 or 2 CRC based on GPER expression (Figure 11A and 11C). Interestingly, male patients with stage 3 or 4 CRC showed no survival difference based on GPER expression (Figure 9D). This patient data indicates a sexual dimorphism of GPER role in CRC progression and survival and is consistent with our *in vitro* studies demonstrating a pro-tumorigenic role for estrogen, acting via GPER, in a hypoxic environment.

## DISCUSSION

This study presents the first analysis of combined estrogen and hypoxia-mediated effects in variously differentiated CRC cell lines. In particular, we have focused on the regulation of pro-angiogenic HIF1-α and VEGFA expression and the regulation of the DNA repair gene *ATM*. Our data demonstrates that E2, acting through GPER, up-regulates HIF1-α and VEGFA expression and promotes proliferation and migration of CRC cells under hypoxic conditions. This finding identifies a potentially pro-tumorigenic role for E2 in the hypoxic environment of ER-negative colorectal tumors.

Previous studies in breast cancer cells showed that estradiol induces the expression of *HIF1A*, which is involved in the transcriptional regulation of VEGF [30, 31]. We tested this relationship in a panel of CRC cell lines. Here we show that in normoxic colorectal cancer cells, E2 can repress HIF1-α and VEGFA expression. By contrast, in hypoxic CRC cells, E2 potentiated the hypoxia-induced HIF1-α and VEGFA expression. Thus estrogenic responses appear to be both cell type-specific and dependent on the cellular hypoxic microenvironment. Moreover, our results corroborate previous studies in hypoxic embryonic cells [31], where *HIF1A* mediates transcriptional activation of the *VEGF* gene and E2 enhances the expression of *HIF1A* and *VEGF*.

GPER has previously been reported as a target of HIF1-α in breast cancer cells [32] and here we confirm that hypoxia induces the expression of GPER in CRC cells. Given that ERβ is frequently lost in the hypoxic microenvironment as CRC malignancy progresses, this up-regulation of GPER is important to maintain estrogenic signaling. Furthermore, we demonstrate that E2 itself enhances GPER expression under both normoxic and hypoxic conditions, enabling an enhanced estrogenic response. It is well established that selective estrogen receptor ligands can produce differential effects by acting on different receptors within the one cell. Here we have demonstrated that in the absence of ERα or ERβ, GPER is essential for transducing both the protective effects (in normoxia) and pro-tumorigenic effects (in hypoxia) of E2. Thus in order to fully understand an estrogenic response it is essential to appreciate not only the estrogen receptor status of the tumor cells but also the hypoxic conditions of the local tumor microenvironment.

*ATM* was identified as a novel E2-repressed mRNA in HT-29, HCT116 and DLD-1 CRC cells and, interestingly, was also variously repressed under hypoxia in these CRC cell lines. The E2-mediated repression of *ATM* was found to occur via GPER-mediated signalling. This is in contrast to the ERα-mediated repression of *ATM* found in breast cancer cells [33]. Though not explored in this study, it is conceivable that other mechanisms of E2 action, in addition to activation of GPER, may contribute to the down-regulation of *ATM* expression. Estrogen increased GPER mRNA and protein expression under both normoxic and hypoxic conditions while E2 had a more potent repressive effect on ATM expression under hypoxic conditions. The regulatory effect of E2 on the expression of GPER may explain some differences in the potency of E2 and G1 to modulate ATM expression. For example, the GPER agonist G1 repressed *ATM* expression to a lesser extent than E2. However, owing to the undetectable expression of ERα in HT-29 cells, this data clearly highlights that different mechanisms for E2-mediated *ATM* repression occur in a cell type-specific manner. Since the loss of *ATM* expression is associated with worse prognosis in CRC patients [22] and ATM expression is reduced in colonic adenomas [34], further repression by combined E2 and low oxygen tension may enhance tumorigenesis.

E2 is frequently considered to play a protective role in CRC. This is evidenced by epidemiological studies in premenopausal women, or postmenopausal women taking hormone replacement therapy (HRT), who are significantly less likely than males to develop CRC [1, 36]. Moreover, a better CRC survival of women compared to men is found especially in the younger age groups [2, 35]. The cellular basis for this protective effect of E2 is believed to be through ERβ and ongoing research is exploring the possibility of utilizing SERMs as a form of treatment in CRC [36]. Our findings indicate that E2 may also have a pro-tumorigenic role in certain hypoxic microenvironments (for example, deep within a tumor), emphasizing the importance of research into GPER-specific ligands in CRC treatment for ERβ negative patients and in hypoxic tumors.

## MATERIALS AND METHODS

### Cell culture and treatments

Colon cancer cells (HT-29, HCT116, DLD-1 and HT55) and rectal cancer cells (SW837, C80 and C99) were a kind gift from Professor Ian Tomlinson (Wellcome Trust Centre for Human Genetics, Oxford, UK). We hold an extensive database for cross-checking CRC cell lines including karyotype, *APC*, *KRAS*, *SMAD4* and *TP53* mutation, microsatellite instability status and presence/absence of 18q. Cells were cultured in Dulbecco's Modified Eagle's Medium (DMEM) (Sigma-Aldrich) supplemented with 10% foetal bovine serum, 2mM L-glutamine and 1% penicillin/streptomycin stock solution (Gibco BRL/Life Technology Inc.) at 37°C, 5% carbon dioxide and 20.9% oxygen. For low oxygen tensions, cells were grown in an Invivo2 1000 Hypoxia Workstation (Ruskinn Life Sciences Ltd.) at 1-2% oxygen (hypoxic) for 24, 48 or 72h. Cell culture media was replaced with serum- and phenol red-free DMEM (Sigma) overnight prior to treatment with E2 (10nM) (Sigma) or ethanol (vehicle) for 24h. For siRNA transfection experiments, HT-29 cells were incubated 48h post transfection under normoxic (20.9% oxygen) or low oxygen tension (1-2% oxygen) in oxygen Control Glove Box Hypoxic Chamber (Coy Lab. Products) and treated with E2 (10nM), vehicle (EtOH) or phenol red-free DMEM for 24h.

### Reverse transcription and real-time quantitative PCR

Total RNA was extracted using Total RNA Purification Kit® (Norgen, Biotek Corp.) Reverse transcription was performed using the High Capacity RNA-to-cDNA™ Kit (Applied Biosystems®). The TaqMan® assays used were: *HIF1A*: Hs00936366_m1; *VEGFA*: Hs99999070_m1; *GPER*: Hs01922715_s1; *ATM*: Hs01112307_m1. Real-time quantitative PCR analyses were performed in triplicate on a 7900 HT Fast Realtime System. Peptidylprolyl isomerase B (cyclophilin B) (*PPIB*) (Hs00168719_m1) [37] or 18S rRNA (Hs99999901_s1) were used for normalization. A relative fold change in expression of the target gene transcript was determined using the comparative cycle threshold method (2^-ΔΔCT^) [38]. A Ct value of 35 represents single molecule template detection, and so where Ct values are ≥35 the target gene is considered not expressed [39].

### Western blot analysis

Protein cell lysates were prepared by washing cells in phosphate-buffered saline (PBS) twice and solubilizing with RIPA buffer. Protein concentration in the supernatant was determined using the DC™ Protein Assay Kit (Bio-Rad Laboratories Inc.). Proteins were separated on 6% (ATM) and 10% (GPER, VEGFA, HIF1-α, N-cadherin, E-cadherin) polyacrylamide gels, blotted onto PVDF membrane and probed with well-characterised antibodies to hGPER (R&D Systems, AF5534, 1:1500 or Abcam Anti-G-protein coupled receptor 30 antibody - C-terminal, ab154069, 1:1500), HIF1-α (BD Transduction Laboratories, 610958, 1:1500), VEGFA (ABS82, Millipore, 1:1500), ATM (Cat. A300-299A, Bethyl, 1:1000), N-Cadherin (sc-59987, Santa Cruz), E-Cadherin (sc-21791, Santa Cruz, 1:1000), GAPDH (A5316, Santa Cruz, 1:5000) and β-actin (Sigma, A5316, 1:10000). Signal was detected using the Amersham™ ECL™ Prime Western Blotting Detection Reagent (GE Healthcare). Image was developed using Amersham Imager 600 and processed using Image J software [40]. As detailed in the data sheets for the antibodies used, GPER is routinely seen by Western blot as a clear band at approximately 55kDa (https://www.rndsystems.com/products/human-gper-antibody_af5534#ds_image_0; http://www.abcam.com/g-protein-coupled-receptor-30-antibody-c-terminal-ab154069.html).

### Cell migration analysis

HT-29 and DLD-1 cell migration was measured by the wound closure rate of an injured confluent cell monolayer seeded at equal densities in 6-well plates. The monolayer, in phenol red-free DMEM containing 10% charcoal-stripped serum (steroid-deprived) was scratched (central vertical line) using a 10μl pipette tip and images were immediately acquired (5 pictures/well, 0h) using a Samsung I310W camera. The medium was replaced with, untreated or treated with E2 (10nM) or G1 (1μM) or G15 (5μM) or vehicle (ethanol 1000x) and incubated at the required oxygen tension for 24h. After incubation, images corresponding to locations at 24h were acquired. Images were obtained from 5 pictures per well using a total of 6 wells per condition performed in triplicate. The fractional closure was measured using Image J software.

### Cell proliferation analysis

HT-29 and DLD-1 wild type and GPER-1 knock down cells were seeded in 96-well plates (5×10^3^ HT-29 cells/well and 1×10^3^ DLD-1 cells/well) and incubated overnight at 37°C. Cells were then either untreated or treated with E2, G1, G15 or vehicle in phenol red-free serum-free medium and incubated at 20.9% or 2% oxygen for 48h. Cell assays were performed using the CellTitre 96® Aqueous One Solution Cell Proliferation Assay (Promega Corporation). Values were normalized to the vehicle-treated normoxic in the case of WT experiments or vehicle-treated normoxic non-transfected control in the case of GPER-knockdown cells at 48h.

### Boyden chamber assay

HT-29 and DLD-1 cells (1.25 × 10^5^) were allowed to migrate for 48 h through 8 μm pore cell culture inserts (24 well Millicell, Millipore). Non-migratory cells were carefully removed by Q-tip from the surface of the inserts. Migratory cells were stained with 0.1% crystal violet (Crystal violet 1% aqueous solution, V5265, Sigma). Three independent fields of migratory cells per well were photographed under phase contrast microscopy. To quantify invasive cells, acetic acid 30% was used to remove the crystal violet and then absorbance was measured at 490nm. Each condition of migration assay was repeated on 3 independent occasions.

### Transfection with GPER1 siRNA

HT-29 cells and DLD-1 were seeded in 5ml phenol red-free DMEM (Sigma) growth medium into 25cm^2^ culture flasks and grown to 80% confluence. The cells were washed in PBS twice, trypsinized, spun down at 1100g and re-suspended in culture medium. *Silencer*® Select Negative Control #1 siRNA (5nmol, Cat.#AM4635, Ambion®), GPER-1 Silencer selected RNA (5 nmol, Cat.# 4390824, Ambion®) were introduced into the cells by reverse transfection using the siPORT™ NeoFX™ transfection reagent (Ambion®). In brief, the siRNA was dissolved in RNase-free water at a concentration of 50μM and then 100nM of each siRNA stock solution was diluted in 100μl of OptiMEM medium (Sigma Aldrich). Separately, 5μl of siPORT™ NeoFX™ transfection reagent was dissolved in 100μl of OptiMEM medium. Equal volumes of the diluted siRNA and transfection reagent were mixed and incubated at room temperature for 10min. The cells were prepared in 1ml of OptiMEM medium at 3×10^4^ density in six-well plates for mRNA and protein analysis. The mixture of siRNA and transfection reagent was added to each well and the transfection was started. After 6h, 1ml of a phenol red-free DMEM (Sigma) containing 10% of FBS and antibiotics was added to the transfected cells. After further 18h, the remaining siRNA was aspirated and cells were grown in serum-free and phenol red-free DMEM (Sigma) for 24h.

### Kaplan-Meier analysis

Kaplan-Meier analysis of relapse free patient survival was carried out in 566 primary colon cancer tumors using the R2 bioinformatics web tool (R2: Genomics analysis and visualization platform http://r2.amc.nl) on the previously published data set GSE39582 [41] Substratification of tumors based on patient gender and tumor stage at diagnosis was also carried out. Median gene expression was used to stratify patients into high or low expression for the gene being examined. P-values were calculated based on a log rank test.

### Statistical analyses

All values are reported as means ± SEM and compared using a Student's *t*-test. To compare multiple groups, a one-way analysis of variance (ANOVA) multiple comparisons test was applied. Kruskal-Wallis nonparametric test was applied to compare significance between two or more groups, as appropriate. Differences between the means were considered statistically significant when p≤0.05.

## CONCLUSION

This study highlights the influence of GPER status and the cellular hypoxic microenvironment on E2 action in CRC malignancy. E2 treatment synergises with hypoxia by repressing *ATM* expression via GPER, and combined with the activation of HIF1-α and VEGFA, potentiates the hypoxia-induced cell migration and proliferation. It appears that the role of E2 in CRC progression is complicated by the relative expression of the different estrogen-ligand receptors and GPER under varying ambient oxygen tension and is not simply, as previously suggested, to be a unique protective effect through ERβ activation. The potential exacerbating effects of E2 in CRC under hypoxic conditions and its signal transduction via GPER require further research. The sexual dimorphism of E2 actions on CRC cell biology transduced through differential ER/GPER expression under varying oxygen tensions may resolve the controversies of epidemiological studies confounded by age, gender, hypoxia, tumor stage and HRT in CRC patients.

## SUPPLEMENTARY MATERIALS FIGURES


