# Supplementary material for: GPER mediates differential effects of estrogen on colon cancer cell proliferation and migration under normoxic and hypoxic conditions
Source: Oncotarget. 2017 Sep 6;8(48):84258–75. doi: 10.18632/oncotarget.20653 (PMC5663593; doi:10.18632/oncotarget.20653)
Supplement: Supplementary file 1 [file oncotarget-08-84258-s001.pdf]

GPER mediates differential effects of estrogen on colon cancer cell proliferation and migration under normoxic and hypoxic conditions

SUPPLEMENTARY MATERIALS

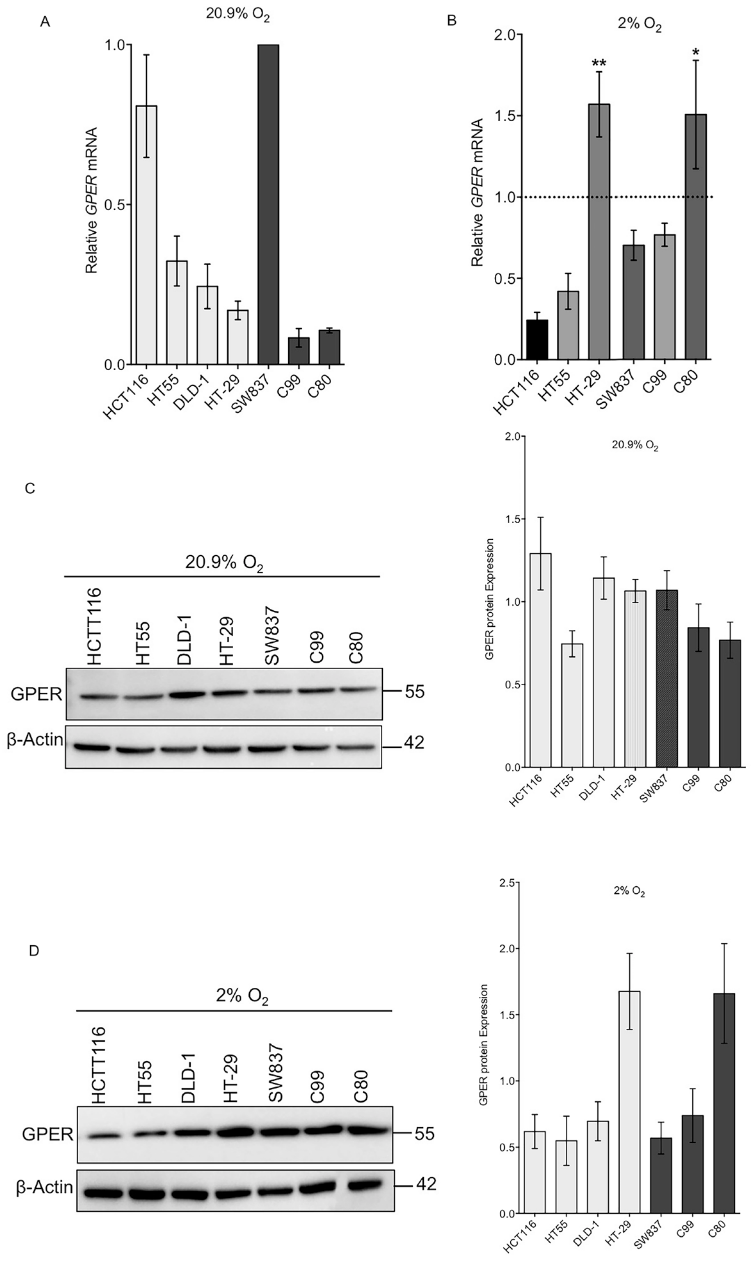

Supplementary Figure 1: Hypoxia increases mRNA and protein GPER-1 expression in moderately well differentiated CRC cell lines. Expression of GPER in colon (HCT116, HT55, DLD-1 and HT-29) and rectal (SW837, C99 and C80) cancer cell lines cultured in 20.9% oxygen **(A)** or in 2% oxygen (hypoxic) **(B)** for 24h. Bars show relative change in GPER expression in each cell line under normoxic conditions relative to the highest GPER expresser SW837 and in hypoxic conditions relative to the respective oxic control (expression value of 1). Mean ± SEM, *n*=3-4, $^{*}P<0.01$, $^{**}P<0.001$. GPER mRNA levels were normalized to PPIB endogenous control. Western Blot and densitometry analysis of GPER protein expression in colon (HCT116, HT55, DLD-1 and HT-29) and rectal (SW837, C99 and C80) cancer cell lines cultured in 20.9% oxygen **(C)** and 2% oxygen **(D)** for 24h. Mean ± SEM, *n*=4, $^{**}P=0.002$.

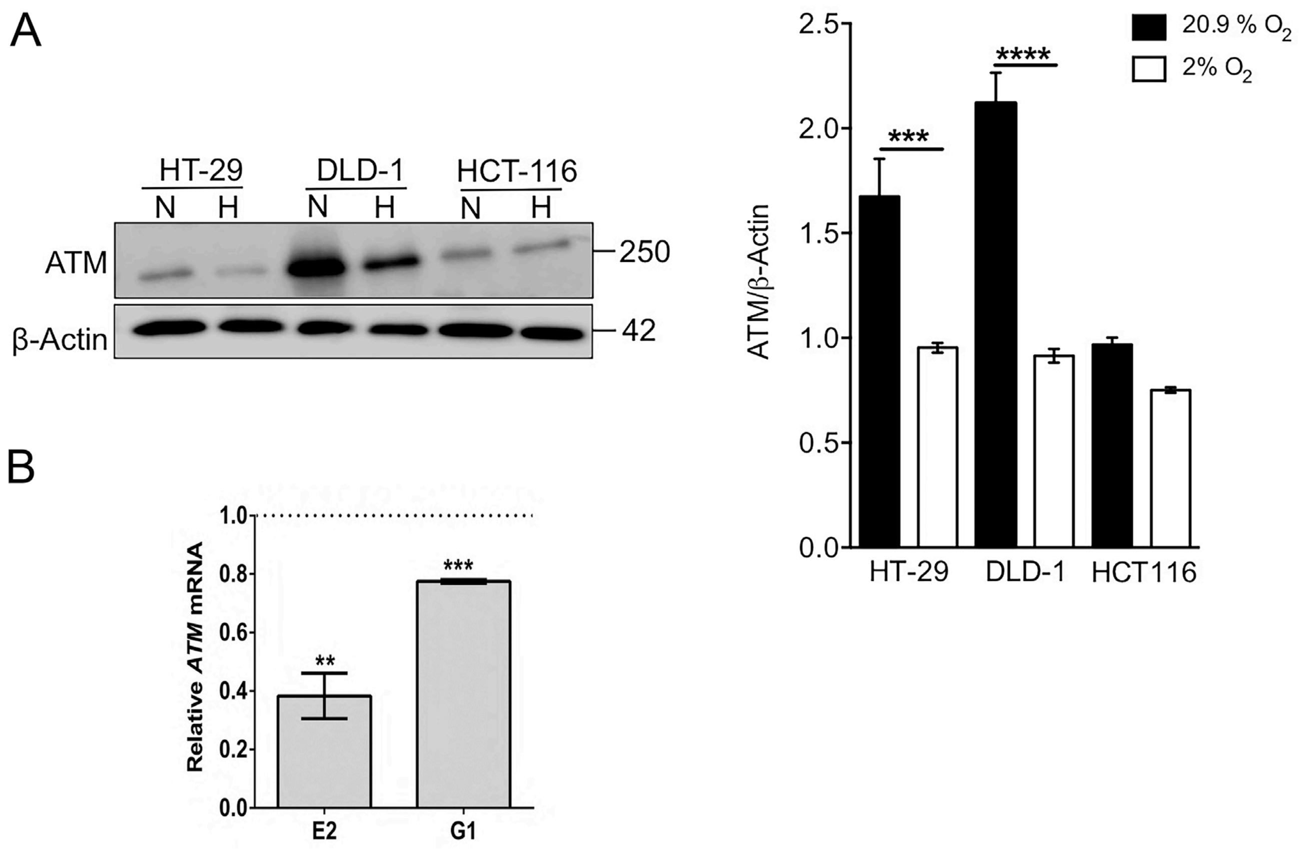

Supplementary Figure 2: Hypoxia induces expression of ATM in colon cancer cells. (A) Western Blot and densitometry analysis of ATM in a panel of colon (HT-29, DLD-1 and HCT116) cancer cell lines of varying differentiation, cultured in normoxic and hypoxic conditions for 24h. β-actin was used as a loading control. Mean ± SEM, *n*=4. $^{***}P<0.001$, $^{****}P<0.0001$. **(B)** Expression values ascertained by RT-qPCR for *ATM*, normalized to 18S rRNA endogenous control, in HT-29 colon cancer cells treated with vehicle (ethanol or DMSO), E2 (10nM) or G1 (1µM) for 24 h. Graphs show relative change in mRNA expression under treatment conditions relative to the vehicle control (mean ± SEM), which was assigned an expression value of 1. $^{**}P<0.01$, $^{***}P=0.008$.

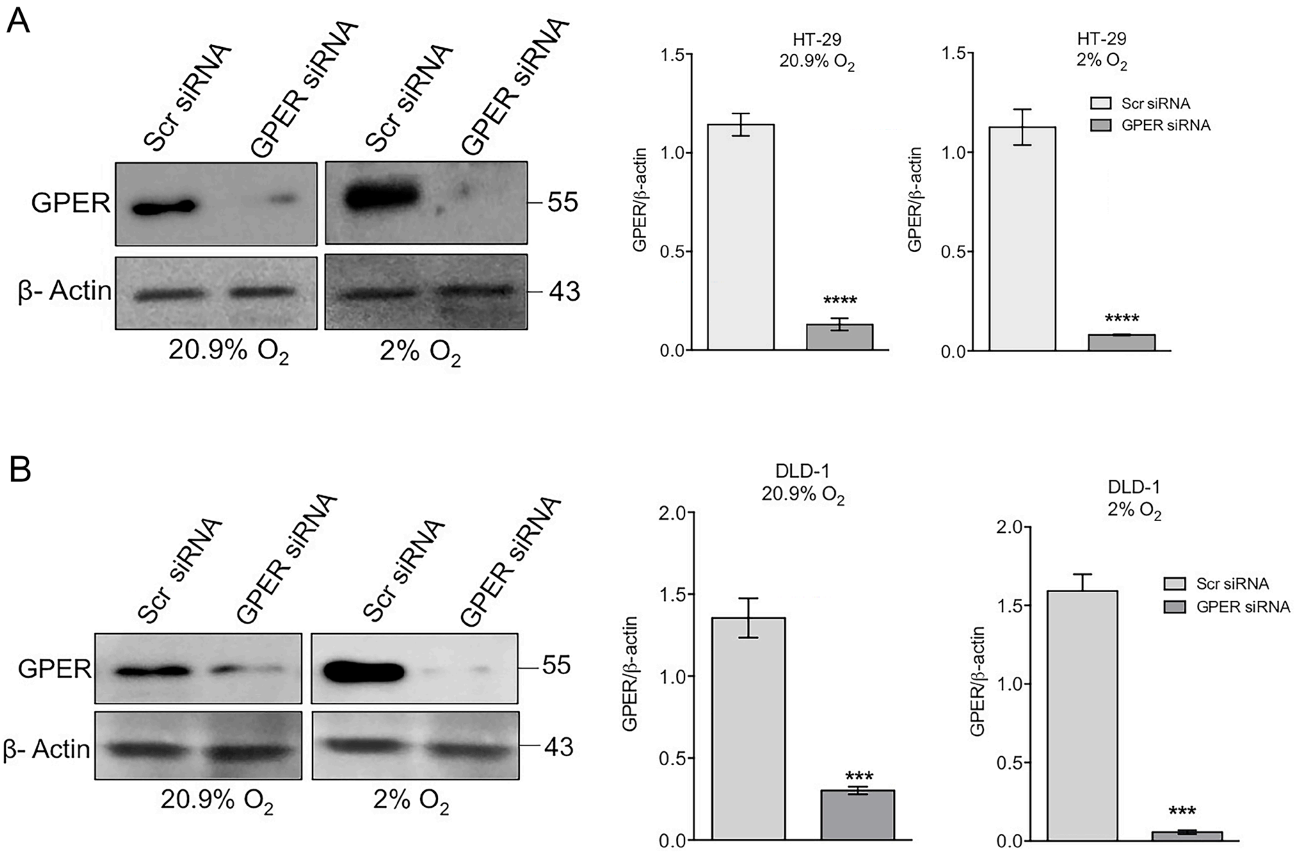

Supplementary Figure 3: GPER knockdown using GPER siRNA in HT-29 and DLD-1 cell lines. **(A)** Western blot and densitometry analysis confirming GPER knockdown using GPER siRNA in HT-29 cells under normoxic and hypoxic conditions. Mean ± SEM, n=4. ****P<0.0001. **(B)** Western blot and densitometry analysis confirming GPER knockdown using GPER siRNA in DLD-1 cells under normoxic and hypoxic conditions. Mean ± SEM, n=4. ***P<0.001.
